# Comparison of Spinal Cord Magnetic Resonance Imaging Features Among Children With Acquired Demyelinating Syndromes

**DOI:** 10.1001/jamanetworkopen.2021.28871

**Published:** 2021-10-13

**Authors:** Giulia Fadda, Cesar A. Alves, Julia O’Mahony, Denise A. Castro, E. Ann Yeh, Ruth Ann Marrie, Douglas L. Arnold, Patrick Waters, Amit Bar-Or, Arastoo Vossough, Brenda Banwell

**Affiliations:** 1 Montreal Neurological Institute, McGill University, Montreal, Quebec, Canada; 2 Division of Neuroradiology, Department of Radiology, Children’s Hospital of Philadelphia, Philadelphia, Pennsylvania; 3 Department of Internal Medicine, Max Rady College of Medicine, Rady Faculty of Health Sciences, University of Manitoba, Winnipeg, Manitoba, Canada; 4 Department of Community Health Sciences, Max Rady College of Medicine, Rady Faculty of Health Sciences, University of Manitoba, Winnipeg, Manitoba, Canada; 5 Department of Diagnostic Radiology, Queen’s University, Kingston, Ontario, Canada; 6 Department of Pediatrics, University of Toronto, Toronto, Ontario, Canada; 7 Nuffield Department of Clinical Neurosciences, John Radcliffe Hospital, University of Oxford, Oxford, United Kingdom; 8 Center for Neuroinflammation and Neurotherapeutics, Multiple Sclerosis Division, Department of Neurology, Perelman School of Medicine, University of Pennsylvania, Philadelphia; 9 Division of Child Neurology, Department of Neurology, The Children’s Hospital of Philadelphia, Perelman School of Medicine, University of Pennsylvania, Philadelphia

## Abstract

**Importance:**

The recognition of magnetic resonance imaging (MRI) features associated with distinct causes of myelitis in children is essential to guide investigations and support diagnostic categorization.

**Objective:**

To determine the clinical and MRI features and outcomes associated with spinal cord involvement in pediatric myelin oligodendrocyte glycoprotein antibody–associated disease (MOGAD), multiple sclerosis (MS), and seronegative monophasic myelitis.

**Design, Setting, and Participants:**

In this cohort study, participants were recruited between 2004 and 2017 through the multicenter Canadian Pediatric Demyelinating Disease Study, which enrolled youth younger than 18 years presenting within 90 days of an acquired demyelinating syndrome. Of the 430 participants recruited, those with lesions on available spine MRI and anti-MOG testing performed on archived samples obtained close to clinical presentation were selected. Participants with poor-quality images and final diagnoses of nondemyelinating disease, anti–aquaporin 4 antibody positivity, and relapsing seronegative myelitis were excluded. Data analysis was performed from December 2019 to November 2020.

**Main Outcomes and Measures:**

Spinal cord involvement was evaluated on 324 MRI sequences, with reviewers blinded to clinical, serological, and brain MRI findings. Associated clinical features and disability scores at 5 years of follow-up were retrieved. Results were compared between groups.

**Results:**

A total of 107 participants (median [IQR] age at onset, 11.14 [5.59-13.39] years; 55 girls [51%]) were included in the analyses; 40 children had MOGAD, 21 had MS, and 46 had seronegative myelitis. Longitudinally extensive lesions were very common among children with MOGAD (30 of 40 children [75%]), less common among those with seronegative myelitis (20 of 46 children [43%]), and rare in children with MS (1 of 21 children [5%]). Axial gray matter T2-hyperintensity (ie, the H-sign) was observed in 22 of 35 children (63%) with MOGAD, in 14 of 42 children (33%) with seronegative myelitis, and in none of those with MS. The presence of leptomeningeal enhancement was highly suggestive for MOGAD (22 of 32 children [69%] with MOGAD vs 10 of 38 children [26%] with seronegative myelitis and 1 of 15 children [7%] with MS). Children with MOGAD were more likely to have complete lesion resolution on serial images (14 of 21 children [67%]) compared with those with MS (0 of 13 children).

**Conclusions and Relevance:**

These findings suggest that several features may help identify children at presentation who are more likely to have myelitis associated with MOGAD. Prominent involvement of gray matter and leptomeningeal enhancement are common in pediatric MOGAD, although the pathological underpinning of these observations requires further study.

## Key Points

**Question:**

What features distinguish spinal cord involvement in children with myelin oligodendrocyte glycoprotein antibody–associated disease (MOGAD), multiple sclerosis (MS), and seronegative monophasic myelitis?

**Findings:**

In this cohort study of 107 children with acquired demyelinating syndromes, features distinguishing children with MOGAD from those with MS and seronegative myelitis included longitudinally extensive spinal cord lesions, complete cross-sectional involvement, H-sign, and leptomeningeal enhancement. Myelitis in MOGAD often demonstrates substantial clinical and radiological resolution; longitudinally extensive lesions were rare in children with MS and less frequent than previously reported.

**Meaning:**

These findings suggest that MOGAD-associated myelitis is characterized by prominent gray matter and leptomeningeal involvement, features that may serve as useful diagnostic clues.

## Teaser

This cohort study examines the clinical and magnetic resonance imaging (MRI) features and outcomes associated with spinal cord involvement in pediatric myelin oligodendrocyte glycoprotein antibody–associated disease, multiple sclerosis, and seronegative monophasic myelitis.

## Introduction

Spinal cord involvement is common among several pediatric acquired demyelinating syndromes.^1^ Magnetic resonance imaging (MRI) is a key element of the diagnostic workup and is often combined with testing for central nervous system (CNS)–targeted antibodies. In addition to providing initial diagnostic clues, the recognition of characteristic imaging features is essential when such antibody testing is not readily available, results are borderline, or serological testing occurs well after the acute illness or following treatments, such as plasma exchange, that may alter diagnostic yield.

The available literature delineating imaging features of spinal cord lesions in children with multiple sclerosis (MS) and other acquired demyelinating diseases largely predates the availability of testing for antibodies to myelin oligodendrocyte glycoprotein (MOG). Because MOG antibodies are now known to be present in approximately one-third of children with acute demyelination,^2,3,4,5^ it is timely to reevaluate neuroimaging features in light of MOG serostatus. This reevaluation is of particular interest in children with longitudinally extensive transverse myelitis (LETM) because LETM has been associated with neuromyelitis optica with antibodies to aquaporin 4 (AQP4) and acute flaccid myelitis and has been reported in approximately 15% of children with a diagnosis of MS.^6,7,8^ Data comparing the relative frequency of LETM in children with MOG antibody–associated disease (MOGAD), MS, and seronegative monophasic demyelination are limited.^9^ We provide a systematic and in-depth imaging characterization of spinal cord lesions in children with MOGAD, MS, and monophasic seronegative myelitis enabled by a multicenter longitudinal prospective study of children with incident CNS demyelination.

## Methods

### Participants

This cohort study was approved by the institutional review boards of all participating institutions. Parents or guardians and older participants provided written informed consent. Younger children provided verbal assent. This study follows the Strengthening the Reporting of Observational Studies in Epidemiology (STROBE) reporting guideline for observational studies.

Between 2004 and 2017, the Canadian Pediatric Demyelinating Disease Study recruited 430 children and adolescents with presumed acquired demyelinating syndromes who presented at any of the 24 participating pediatric clinics. The research protocol included clinical neurological examination, acquisition of serological samples, and brain MRI scans at presentation and prospectively at 3, 6, and 12 months and yearly thereafter. Cerebrospinal fluid analysis and spine MRI were acquired whenever indicated for clinical care.

All participants enrolled in the Canadian Pediatric Demyelinating Disease Study for whom spinal cord MRI was performed as part of their clinical care were eligible for this study. For inclusion, participants had to have spinal cord lesions detected on MRI and either positive serological findings for anti-MOG antibodies or negative results on serum obtained within 45 days from presentation. Comparisons of clinicoradiological features were performed between participants with 1 of the 3 confirmed diagnoses (MS,^10^ MOGAD, or seronegative monophasic myelitis). We therefore excluded 1 child with anti-AQP4 antibodies, 8 children with a final diagnosis of nondemyelinating disease, and 1 child with relapsing myelitis without MOG or AQP4 antibodies and without brain MRI lesions.

### MRI Acquisition and Analysis

A total of 324 spine MRI studies, including axial and sagittal T2-weighted and unenhanced and gadolinium-enhanced T1-weighted sequences, were evaluated; 246 scans showed spinal cord lesions and were considered in the final analysis. A spinal cord MRI scoring tool was designed on the basis of existing literature^11^ and authors’ experience (eAppendix in Supplement 1). After training on the scoring tool, a neuroradiologist (C.A.A.) and a neurologist with neuroimaging expertise (G.F.) evaluated all eligible images for the presence, location, and appearance of spinal cord lesions. The neuroradiologist (C.A.A.) applied the scoring tool for the description of the pattern of gadolinium enhancement. A senior neuroradiologist (A.V.) served to adjudicate cases of uncertainty or disagreement between scorers. The scoring was performed with the reviewers blinded to clinical, serological, and brain MRI results. All brain MRI scans acquired as part of the study protocol had been independently evaluated for the presence of lesions.^12^

### Clinical Features

For each participant, demographic (age, sex, and ethnicity) and clinical data, including the phenotype of presenting and subsequent attacks (eg, optic neuritis, myelitis, and acute disseminated encephalomyelitis [ADEM]),^13^ were recorded. Ethnicity was defined by the investigator and was assessed in this study because of previous reports of ethnicity-related phenotype differences in patients with MS and neuromyelitis optica spectrum disorder.^14,15^ Disability score was extrapolated from detailed neurological examination reports to create an estimated Expanded Disability Status Scale score, per our previously published methods.^16^ To estimate the clinical outcome at equivalent time from presentation in all participants, we selected the Expanded Disability Status Scale score at 5 years after onset (median duration of follow-up).

### Serum Antibody Testing

All serum samples were processed centrally and archived, and then retrieved, batch-shipped, and analyzed in a single laboratory using a live cell–based MOG-IgG1–specific assay, blinded to clinical details.^17^ AQP4 antibodies were also measured using a live cell–based assay. None of the samples was obtained after plasma exchange.

### Statistical Analysis

Demographic, clinical, and MRI findings were compared between participants with spinal cord lesions in the MOGAD group and each of the seronegative groups (MS and seronegative myelitis) using 2-sided χ^2^ or Fisher exact tests for categorical variables and Mann-Whitney *U* tests for continuous variables. Features were also compared between MOGAD children with or without ADEM phenotype. The analyses were performed using Python statistical software version 3.6.5 (Python). Data analysis was performed from December 2019 to November 2020.

## Results

### Participants

**Figure 1.**
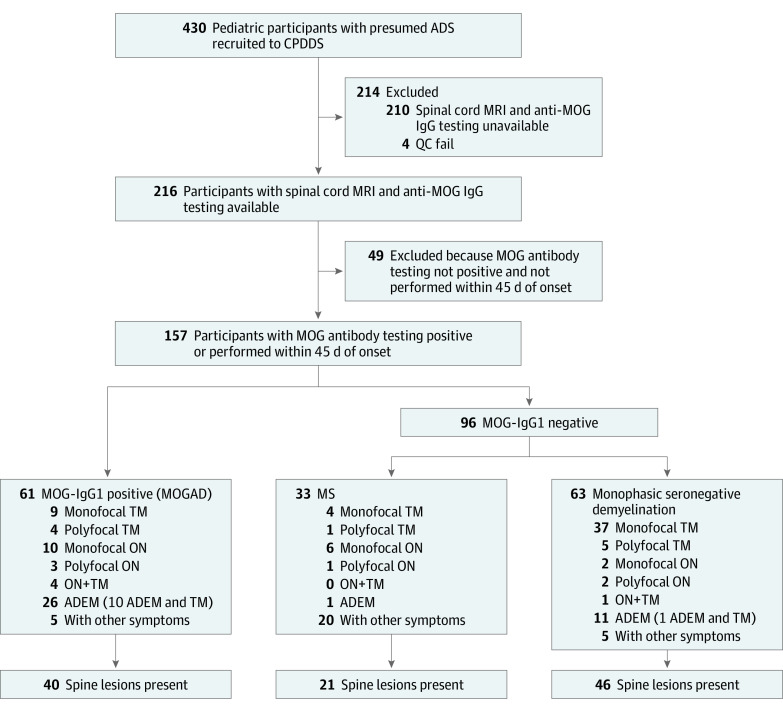
Flowchart of Study Design and Patient Recruitment ADEM indicates acute disseminated encephalomyelitis; ADS, acquired demyelinating syndrome; CPDDS, Canadian Pediatric Demyelinating Disease Study; MOG-IgG, myelin oligodendrocyte glycoprotein antibodies; MOGAD, myelin oligodendrocyte glycoprotein antibody–associated disease; MRI, magnetic resonance imaging; MS, multiple sclerosis; ON, optic neuritis; QC, quality control; TM, transverse myelitis.

Of the 157 participants with spine MRI available, 107 had spinal cord lesions (40 with MOGAD, 21 with MS, and 46 with seronegative myelitis) (Figure 1). The median (IQR) age at onset of these 107 participants was 11.14 (5.59-13.39) years, and 55 (51%) were girls (Table 1). Children with MOGAD were significantly younger than those with MS (median [IQR] age at onset, 6.71 [3.80-11.48] vs 14.00 [13.01-14.67] years; odds ratio, 0.016 [95% CI, 0.0-0.1]), whereas no differences were detected in the frequency of reported ethnicities across the 3 groups.

**Table 1.** Clinical Features of Participants With Spinal Cord Lesions on MRI

Characteristic	Participants, No. (%)			OR (95% CI)	
	MOGAD (n = 40)	MS (n = 21)	Seronegative myelitis (n = 46)	MOGAD vs MS	MOGAD vs Seronegative myelitis
Sex					
Female	23 (57)	14 (67)	18 (39)	0.68 (0.2-2.0	2.1 (0.9-5.0)
Male	17 (43)	7 (33)	28 (61)		
Age at onset, median (IQR), y	6.71 (3.80-11.48)	14.00 (13.01-14.67)	11.12 (6.23-12.88)	0.016 (0.0-0.1)	0.25 (0.1-0.6
Ethnicity					
African	1 (2)	1 (5)	1 (2)	0.51 (0.0-8.6)	1.2 (0.1-19.1)
Asian	5 (12)	2 (10)	6 (13)	1.4 (0.2-7.7)	0.95 (0.3-3.4)
European	18 (45)	12 (57)	25 (54)	0.61 (0.2-1.8)	0.69 (0.3-1.6)
Mixed or other^a^	15 (38)	6 (29)	10 (22)	1.5 (0.5-4.7)	2.2 (0.8-5.6)
Unknown	1 (2)	0	4 (9)	1.63 (0.06-41.83)	0.27 (0.0-2.5)
Time from first clinical presentation to serum sampling, median (IQR), d	18 (8-31)	11 (7-22)	5 (4-12)	2.0 (0.7-6.0)	4.8 (1.9-12.0)
Time from first clinical presentation to acquisition of first MRI with lesions, median (IQR), d	9 (4-18)	68 (10-749)	6 (2-7)	0.24 (0.1-0.8)	2.8 (1.2-6.7)
Phenotype at time of MRI^b^					
Transverse myelitis	11 (28)	4 (19)	35 (76)	1.6 (0.4-5.9)	0.12 (0.1-0.8)
Acute disseminated encephalomyelitis	10 (25)	0	6 (13)	14.8 (0.02-266.4)	2.2 (0.7-6.8)
Acute disseminated encephalomyelitis and transverse myelitis	7 (18)	0	1 (2)	9.62 (0.52-177.34)	9.5 (1.1-81.4)
Optic neuritis	2 (5)	1 (5)	0	1.1 (0.1-12.3)	NA
Other	6 (15)	6 (24)	3 (7)	0.14 (0.03-0.64)	2.53 (0.59-10.86)
No symptoms in the preceding 30 d	4 (10)	10 (48)	1 (2)	0.12 (0.03-0.47)	5.0 (0.54-46.72)
Episodes of myelitis >30 d before the acquisition of first MRI with lesions	0	2 (10)	0	0.10 (0.0-2.10)	1.15 (0.02-59.18)
Prior negative MRI	1 (3)	2 (10)	1 (2)	0.24 (0.02-2.86)	1.15 (0.07-19.06)
Brain lesions present on MRI	28 (70)	21 (100)	20 (43)	0.0	3.0 (1.2-7.4)

Abbreviations: MOGAD, myelin oligodendrocyte glycoprotein antibody–associated disease; MRI, magnetic resonance imaging; MS, multiple sclerosis; NA, not applicable; OR, odds ratio.

^a^ Other refers to North American Aboriginal origins, other North American origins, and Latin, Central, and South American origins.

^b^ Refers to phenotype of clinical attacks reported in the 30 days preceding the first spine MRI examination with lesions.

The first MRI scan with lesions was acquired within 30 days following an acute clinical attack in 36 of 40 participants (90%) with MOGAD, 11 of 21 participants (54%) with MS, and 45 of 46 participants (98%) with seronegative myelitis. As shown in Table 1, the phenotype of this attack was myelitis (or ADEM with myelitis) in 18 of 40 participants (45%) with MOGAD, 4 of 21 participants (19%) with MS, and 36 of 46 participants (78%) with seronegative myelitis.

The first abnormal spine MRI was the first available scan in 103 of 107 participants, and 4 additional children developed spinal cord lesions on subsequent examinations. Two of them (1 with MOGAD and 1 with seronegative myelitis) had an initial MRI obtained at 1 and 14 days after the first myelitis symptoms, and spinal lesions were detected in the examinations performed 2 days and 4 months from the initial MRI, respectively (eFigure 1 in Supplement 1). The other 2 children with initially normal MRI, both with a diagnosis of MS, never experienced clinical episodes of myelitis but developed asymptomatic spinal cord lesions over the course of follow-up.

Spinal lesions were never detected in 10 children (3 with MOGAD and 7 with seronegative myelitis) who presented with spinal cord symptoms (Figure 1). The first available spinal cord MRI in these cases was obtained after a median (range) of 127 (1-936) days from presentation. Brain MRI lesions were observed in all participants with MS, in 28 of 40 participants (70%) with MOGAD, and in only 20 of 46 participants (43%) with seronegative myelitis (8 of whom presented with isolated myelitis).

### Spinal Cord MRI Findings

Although spinal cord lesions were commonly located in the cervical segments in all groups, lesions involving thoracic segments and the conus medullaris were 1.9 and 2.2 times more frequent in children with MOGAD than in those with MS and not significantly different compared with the seronegative myelitis group (Table 2). Most spinal cord lesions had both gray and white matter involvement, whereas the presence of lesions restricted to the white matter was highly suggestive of MS (13 of 19 children [68%]) compared with only 2 of 35 children (6%) with MOGAD and 6 of 42 children (14%) with seronegative myelitis; involvement of the complete spinal cord cross-section was more common in MOGAD (25 of 35 children [71%]) than in MS (6 of 19 children [32%]) or seronegative myelitis (18 of 42 children [43%]). Among lesions affecting limited portions of the cord cross-section in children with MOGAD, central gray matter involvement was more common than peripheral locations (Table 2).

**Table 2.** Features Comparison in the First Magnetic Resonance Imaging Examination With Lesion

Feature	Participants, No./total No. (%)			OR (95% CI)	
	MOGAD (n = 40)	MS (n = 21)	Seronegative myelitis (n = 46)	MOGAD vs MS	MOGAD vs Seronegative myelitis
Segment imaged, No. (%)					
Cervical	40 (100)	21 (100)	46 (100)	1.88 (0.04-98.30)	0.87 (0.02-44.90)
Thoracic	39 (98)	19 (90)	45 (98)	4.1 (0.3-48.2)	0.87 (0.10-14.30)
Lumbar	39 (98)	17 (81)	43 (93)	9.2 (1.0-88.3)	1.8 (0.2-20.3)
Any axial view	35 (88)	19 (90)	42 (91)	0.7 (0.1-4.2)	0.67 (0.16-2.67)
Total spine lesion count, median (IQR)	1 (1-2)	2 (1-2)	1 (1-1)	0.74 (0.3-2.1)	3.9 (1.5-10.4
Lesion					
Cervical	30/40 (75)	18/21 (86)	30/46 (65)	0.5 (0.1-2.1)	1.6 (0.6-4.1)
Thoracic	31/39 (79)	8/19 (42)	28/45 (62)	5.3 (0.1-2.1)	2.4 (0.9-6.3)
Lumbar	25/39 (64)	5/17 (29)	18/43 (42)	4.3 (1.3-14.7)	2.6 (1.1-6.3)
Any matter involvement					
Gray and white	30/35 (86)	13/19 (68)	33/42 (79)	2.8 (0.7-10.7)	1.6 (0.5-5.4)
Only white	2/35 (6)	13/19 (68)	6/42 (14)	0.028 (0.000-0.200)	0.36 (0.10-1.90)
Only gray	7/35 (20)	0/19	6/42 (14)	10.30 (0.55-190.32)	1.5 (0.5-5.0)
Any complete cross-section	25/35 (71)	6/19 (32)	18/42 (43)	5.4 (1.6-18.2)	3.3 (1.3-8.7)
Any lesion^a^					
Anterior	4/35 (11)	7/19 (37)	9/42 (21)	0.22 (0.10-0.90)	0.47 (0.10-1.70)
Posterior	6/35 (17)	11/19 (58)	16/42 (38)	0.1 (0.0-0.2)	0.34 (0.10-1.00)
Lateral	3/35 (9)	14/19 (74)	12/42 (29)	0.03 (0.00-0.20)	0.23 (0.10-0.90)
Central gray	13/35 (37)	6/19 (32)	12/42 (29)	1.3 (0.4-4.2)	1.5 (0.6-3.9)
Short	15/40 (38)	20/21 (95)	19/46 (41)	0.03 (0.00-0.02)	0.85 (0.40-2.00)
Any longitudinally extensive transverse myelitis	30/40 (75)	1/21 (5)	20/46 (43)	60.0 (7.1-506.0)	3.9 (1.5-9.8)
Any H sign	22/35 (63)	0/19	14/42 (33)	65.00 (3.62-1166.20)	3.4 (1.3-8.7)
Any snake eyes	6/35 (17)	0/19	9/42 (21)	8.59 (0.45-161.39)	0.76 (0.2-2.4)
Any bright spot	0/35	0/19	1/42 (2)	0.55 (0.01-28.78)	0.42 (0.02-10.80)
Any tumefactive lesion	14/40 (35)	3/21 (14)	5/46 (11)	3.2 (0.8-12.9)	4.4 (1.4-13.7)
Any enhancement					
Parenchymal	8/32 (25)	5/15 (33)	7/38 (18)	0.6 (0.2-2.5)	1.5 (0.5-4.6)
Nodular	8/32 (25)	5/15 (33)	7/38 (18)	0.67 (0.20-2.50)	1.5 (0.5-4.6)
Ring	0/32	1/15 (7)	0/38	0.47 (0.01-25.18)	1.18 (0.02-61.38)
Leptom	22/32 (69)	1/15 (7)	10/38 (26)	31.0 (3.5-267.6)	6.2 (2.2-17.4)
Root	11/32 (34)	1/15 (7)	7/38 (18)	7.3 (0.8-63.3)	2.3 (0.8-7.0)

Abbreviations: MOGAD, myelin oligodendrocyte glycoprotein antibody–associated disease; MS, multiple sclerosis; OR, odds ratio.

^a^ Computed among lesions not involving the complete cross-sectional area.

**Figure 2.**
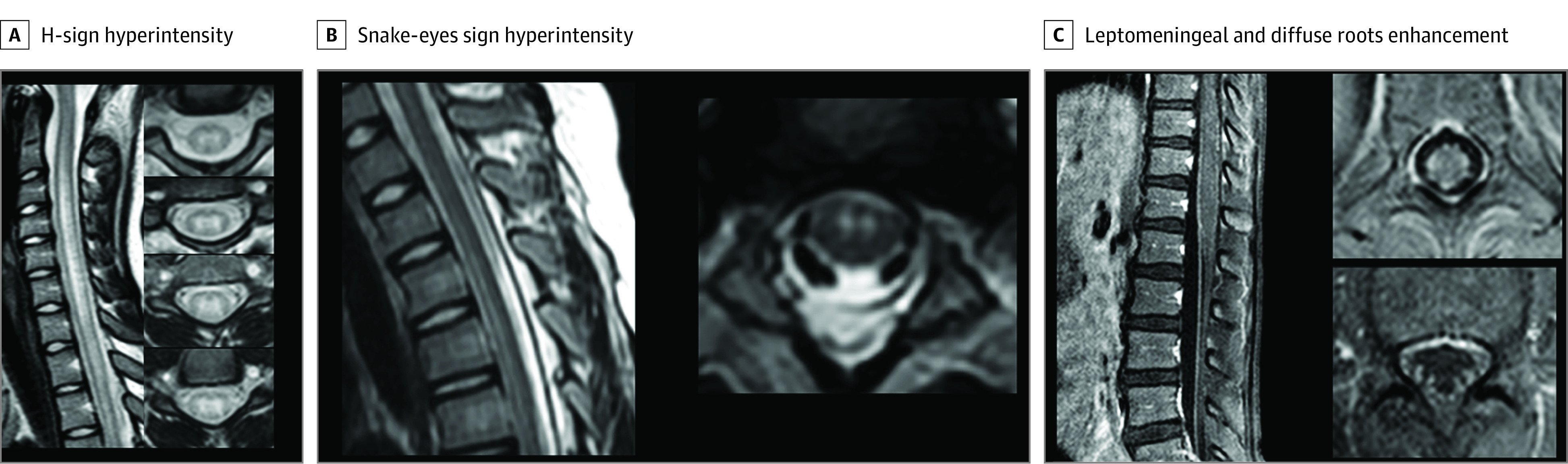
Characteristic Imaging Features of Spinal Cord Involvement in Myelin Oligodendrocyte Glycoprotein Antibody–Associated Disease A, Sagittal (left) and axial (right) T2-weighted magnetic resonance imaging (MRI) of the spine shows a spinal cord lesion spanning over more than 10 vertebral segments, with H-sign appearance most evident in the cranial and caudal extremities of the lesion and hazy involvement of most of the cross-sectional spinal cord area in the central portion of the lesion. B, Sagittal (left) and axial (right) T2-weighted MRI of the spine shows a longitudinally extensive lesion with pencil-sign appearance on sagittal view and corresponding anterior-horns hyperintensity on axial image. C, Sagittal (left) and axial (right) gadolinium-enhanced T1-weighted MRI of the spine shows leptomeningeal and diffuse roots enhancement.

Marked T2-hyperintensity of the gray matter on axial views (ie, the H-sign) was observed in 22 of 35 lesions (63%) in participants with MOGAD (Figure 2A). In fewer cases (6 of 35 cases [17%]), the hyperintensity was restricted to the anterior horns (ie, the snake-eyes sign) (Figure 2B). The T2-hyperintensity of the spine gray matter was often accompanied by fainter hyperintensity in the surrounding white matter, characteristically observed in the central portion of the lesion, whereas gray matter T2-hyperintensity was prominent at the upper and lower extremes. Although the H-sign was never observed among participants with MS, it was found in 14 of 42 participants (33%) with seronegative myelitis. In this latter group, an additional 21% of participants showed the snake-eyes sign. Notably, no bright spotty lesions were detected among children with MOGAD or MS.

Among children with gadolinium-enhanced images available, the frequency of nodular enhancement was similar among those with MOGAD (8 of 32 children [25%]), MS (5 of 15 children [33%]), and seronegative myelitis (7 of 38 children [18%]) (Table 2). The presence of leptomeningeal enhancement among participants with MOGAD (22 of 32 children [69%]) was 9.9 times more frequent than that among those with MS (1 of 15 children [7%]) and 2.7 times more frequent than that among those with seronegative myelitis (10 of 38 children [26%]) (Figure 2C). On axial views, it was characterized by leptomeningeal along with perpendicular linear enhancement, following the distribution of medullary veins (eFigure 2 in Supplement 1). When restricted to participants who did not undergo lumbar puncture and were not exposed to steroids in the 30 days preceding the scan acquisition, the increased frequency of leptomeningeal enhancement remained noticeable in MOGAD (11 of 19 participants [58%]) compared with MS (1 of 8 participants [12%]) and seronegative myelitis (3 of 14 participants [21%]). In addition, 11 of 32 children (34%) with MOGAD, 1 of 15 children (7%) with MS, and 7 of 38 children (18%) with seronegative myelitis demonstrated enhancement of spinal roots (Figure 2C).

### Longitudinally Extensive Transverse Myelitis

Lesions involving 3 or more vertebral segments were common among children with MOGAD (30 of 40 children [75%]) and were observed in 20 of 46 children (43%) with seronegative myelitis. Only 1 of 21 patients (5%) with MS met the criteria for LETM. In this case, the LETM was characterized by a short core of T2-hyperintensity, with associated nodular enhancement and a larger portion of fainter hyperintensity extending over 3 cervical vertebrae (eFigure 3 in Supplement 1). Of note, in 2 participants with MOGAD, 1 with MS, and 7 with seronegative myelitis, lesions that appeared longitudinally extensive on sagittal views were more accurately considered contiguous independent short lesions identifiable on axial planes.

LETM spanning 10 or more vertebral segments were seen in children with MOGAD and seronegative myelitis (eTable 1 in Supplement 1). The presence of leptomeningeal enhancement was particularly frequent among MOGAD children with LETM (19 of 24 children [79%]).

### Serial Spinal Imaging

Serial spine MRI scans were available for 21 of 40 participants (53%) with MOGAD, 13 of 21 participants (62%) with MS, and 29 of 46 participants (63%) with seronegative myelitis (Table 3). Over a median (IQR) of 2.22 (0.69-3.75) years between the first and most recent spine MRI examinations, spinal cord lesions resolved entirely in 14 of 21 children (67%) with MOGAD, for whom complete resolution could be detected as early as 12 days from the initial abnormal MRI. This contrasted with persistence of spinal cord abnormalities in all participants with MS and in 17 of 29 participants (59%) with seronegative myelitis.

**Table 3.** Serial MRI Features and Clinical Outcome

Feature	Participants, No./total No. (%)			OR (95% CI)	
	MOGAD (n = 40)	MS (n = 21)	Seronegative myelitis (n = 46)	MOGAD vs MS	MOGAD vs Seronegative myelitis
Serial spine MRI available	21/40 (53)	13/21 (62)	29/46 (63)	0.68 (0.23-1.20)	0.64 (0.27-1.53)
MRI scans per participant, median (IQR), No.	3 (3-4)	3 (3-4)	2 (2-3)	1.13 (0.30-5.00)	1.92 (0.50-6.90)
Time between first and most recent MRI, median (IQR), y	2.22 (0.69-3.75)	4.24 (3.07-5.30)	1.00 (0.50-3.00)	0.15 (0.00-0.70)	2.30 (0.70-7.30)
Complete lesion resolution^a^	14/21 (67)	0/13	12/29 (41)	32.84 (1.80-590.20)	1.7 (0.7-4.3)
Follow-up ≥5 y available	22/40 (55)	13/21 (62)	22/46 (48)	1.69 (0.66-4.30)	1.33 (0.56-3.11)
Expanded Disability Status Scale score at 5 y from presentation, median (IQR)	1.00 (0.00-1.5)	1.25 (0.5-2.00)	1.50 (0.00-4.00)	0.32 (0.10-1.40)	0.31 (0.10-1.10)
Pyramidal deficits at 5 y from presentation	6/22 (27)	5/13 (38)	14/22 (64)	0.6 (0.1-2.6)	0.21 (0.10-0.80)
Sphincter deficits at 5 y from presentation	1/22 (5)	1/13 (8)	1/21 (5)	0.57 (0.00-10.00)	0.95 (0.10-16.30)

Abbreviations: MOGAD, myelin oligodendrocyte glycoprotein antibody–associated disease; MRI, magnetic resonance imaging; MS, multiple sclerosis; OR, odds ratio.

^a^ Computed among participants with spine lesions and at least 1 month of MRI follow-up.

### Clinical Outcomes

The median (IQR) duration of clinical observation was 5.96 (4.14-8.30) years. At 5 years from presentation, most participants with follow-up data available had favorable outcomes, with median (IQR) Expanded Disability Status Scale scores of 1 (0-1.5) among the 22 participants with MOGAD, 1.25 (0.50-2.00) among the 13 participants with MS, and 1.5 (0.0-4.0) among 22 participants with seronegative myelitis (Table 3). Among the participants with MOGAD whose functional system scores were recorded at 5 years, only 6 of 22 (27%) showed pyramidal signs (all with functional system score of 1), and 1 of 22 (5%) had sphincter dysfunction, whereas pyramidal deficits persisted in 5 of 13 participants (38%) with MS and 14 of 22 participants (64%) with seronegative myelitis; sphincter dysfunction was reported by 1 of 13 participants (8%) with MS and 1 of 21 participants (5%) with seronegative myelitis.

### Imaging Features of Participants With MOGAD With ADEM vs Non-ADEM Phenotypes

Because ADEM is a common MOGAD presentation, we compared spinal MRI findings between children with MOGAD with or without an ADEM phenotype. The key spinal cord features that distinguished MOGAD from MS and seronegative myelitis (H-sign, leptomeningeal enhancement, and complete lesion resolution) were retained (eTable 2 in Supplement 1).

## Discussion

In this cohort study, we performed a comprehensive analysis of 246 spinal MRI scans with lesions obtained from a carefully characterized longitudinal cohort of 107 children with incident CNS demyelination. We highlighted key features of spinal cord involvement in pediatric MOGAD—namely, lesions are often longitudinally extensive and involve lower spine segments, have prominent gray matter involvement (H-sign), and are accompanied by leptomeningeal enhancement. Lesions often involve most of the spinal cord cross-sectional area, in some cases as faded white matter hyperintensity surrounding the H-sign.

Our findings in this incident cohort study both confirm and extend previous observations in adults or in smaller pediatric cohorts.^5,9,18,19,20,21^ Notably, although the presence of the H-sign strongly favors a diagnosis of MOGAD over MS, it is not specific for MOGAD, because it can be found in lesions resulting from infectious and ischemic causes and was observed in 33% of children with seronegative monophasic disease in our cohort. In addition, 17% of children with MOGAD had gray matter T2-hyperintensity restricted to the anterior horns, also resembling infectious or vascular diseases. Despite these worrisome imaging features, most MOGAD-associated spinal lesions resolved, patients did not demonstrate acute flaccid weakness (as is seen in anterior horn lesions associated with enterovirus infections), and few children experienced residual neurological deficits. Bright spotty lesions, which have been reported in 27% to 54% of patients with AQP4 antibodies–associated myelitis,^22,23,24^ were absent in all children with MS and MOGAD in our cohort, underscoring their utility as a distinguishing feature between these diseases.

We report for the first time, to our knowledge, the high frequency (69%) of spinal cord leptomeningeal enhancement in children with MOGAD. Although leptomeningeal enhancement has thus far been sporadically reported in the spinal cord,^25,26,27^ it has been more frequently recognized on brain MRI, with or without associated cortical hyperintensity,^28,29,30,31^ supporting meningeal involvement as a possible key feature of MOGAD.

We also observed spinal root enhancement in 34% of children with MOGAD and in smaller proportions of children with MS and seronegative myelitis. Enhancement of nerve roots is common in children with acute flaccid myelitis^8,9,32^ and could explain the few cases observed in the seronegative-myelitis group. The greater proportion of children with this finding in the MOGAD group is of interest, given the cases of polyradiculopathy in association with anti-MOG antibodies recently reported.^26,33,34,35^ Although our patients did not have frank clinical signs of polyradiculopathy, we cannot exclude the presence of subclinical abnormalities or milder symptoms that were overlooked because of the simultaneous CNS involvement. The mechanism through which antibodies toward MOG, which is exclusively expressed in the CNS, are associated with involvement of the spinal roots remains elusive.

Spinal lesions in pediatric MS are far more likely to occur in the cervical spinal cord, are most commonly eccentric, and involve white matter tracts. Within the MS group, only 19% of children with spinal cord lesions had concurrent myelitis symptoms, emphasizing the value of inclusion of cervical spine images in the evaluation of these patients. Although spinal cord lesions extending up to 3 vertebral segments can be observed in pediatric MS, their frequency in this cohort of children characterized for absence of anti-MOG antibodies is lower than previously reported.^6,7^ Importantly, longer lesions often represent contiguous smaller independent lesions when carefully viewed in axial planes, highlighting the importance of careful assessment of both axial and sagittal images.^36^ The H-sign is not a feature of pediatric MS, and leptomeningeal enhancement is rare.

Seronegative monophasic myelitis, which typically manifests without clinical brain or optic nerve involvement, is longitudinally extensive in almost half of cases, and may involve central or anterior gray matter. The underlying causes are variable. Patients with seronegative myelitis and prominent anterior horns involvement composed 21% of our cohort, and we acknowledge in hindsight that some of these children might have been manifesting acute flaccid myelitis, although this terminology was not commonly used diagnostically at the time of enrollment. In aggregate, of all patients with seronegative monophasic myelitis, 64% were left with some motor dysfunction.

Characterizing the imaging features distinguishing myelitis in MOGAD from other pediatric syndromes has great relevance for clinical practice, particularly given that the serological presence of anti-MOG antibodies is transient in many patients, and, thus, detection of anti-MOG antibodies can be challenging when testing is not performed in proximity to incident demyelination.^2,37^ Even when antibody testing is performed early and anti-MOG antibodies are detected, clinicians should be aware of the possibility of false-positive results, particularly at low titers,^38^ and ultimately should formulate their diagnosis on the basis of the complex of clinicoradiological features. Furthermore, identifying children likely to have had MOG antibodies at onset could be difficult if one were to evaluate spinal cord images obtained weeks from presentation, as evidenced by our analyses of serial images. The nearly complete resolution of most spinal lesions in pediatric patients with MOGAD mirrors findings reported in adult patients with MOGAD^39,40,41^ and is aligned with the similarly high proportion of subjects with resolution of brain T2-hyperintense lesions over follow-up, observed in adult and pediatric cohorts.^2,42^

### Limitations

An inherent limitation of this study is that spine MRI scans were not obtained as part of a formal research protocol but according to clinical indications and with different imaging protocols. Nonetheless, we were able to perform consistent and detailed analyses on scans representing the real-world experience, thus potentially increasing the generalizability of our findings. Given that spinal cord MRI was performed according to clinical indication, it was more likely to be requested for children exhibiting clinical signs of myelitis, and the frequency of asymptomatic lesions detected in our study is possibly underestimated. However, clinical myelitis at the time of MRI acquisition was not a requirement for our study; therefore, the frequency of gadolinium-enhancing lesions is likely reduced compared with studies using images obtained at the time of acute myelitis. This is particularly relevant for the MS group, for whom the time from clinical attack to spine MRI acquisition was longer than for other groups.

## Conclusions

This cohort study identified features of pediatric myelitis that may aid in the identification of specific underlying causes. Most notably, myelitis in MOGAD is typically longitudinally extensive, involving most of the cord cross-sectional and often the lumbar spine (emphasizing the importance of obtaining full-length cord images), can display prominent gray matter hyperintensity, and frequently is associated with leptomeningeal enhancement. These findings are in contrast with those seen in pediatric onset MS, where lesions are typically short but might appear extensive on sagittal views because of confluence of lesions, and involve only a portion of the axial diameter of the cord. Isolated monophasic myelitis has heterogeneous causes and shares imaging features of MOGAD, although it more rarely demonstrates leptomeningeal enhancement.

## Notes

Supplement 1. **eAppendix.** Spine MRI Scoring Tool **eTable 1.** Features Associated With LETM **eTable 2.** Spine Imaging Features of ADEM vs Non-ADEM MOGAD **eFigure 1.** MOGAD With Clinical Presentation of Myelitis and Initial Negative Spine MRI Findings **eFigure 2.** Sagittal and Axial Postgadolinium T1 MR Imaging of the Spine of 5 Different Children With MOGAD Demonstrating Leptomeningeal and Root Enhancement **eFigure 3.** LETM in a 14-Year-Old Girl With MS

Supplement 2.
Canadian Pediatric Demyelinating Disease Study Group

## References

[1] Fadda G, Armangue T, Hacohen Y, Chitnis T, Banwell B. Paediatric multiple sclerosis and antibody-associated demyelination: clinical, imaging, and biological considerations for diagnosis and care. Lancet Neurol. 2021;20(2):136-149. doi:10.1016/S1474-4422(20)30432-4 33484648

[2] Waters P, Fadda G, Woodhall M, ; Canadian Pediatric Demyelinating Disease Network. Serial anti-myelin oligodendrocyte glycoprotein antibody analyses and outcomes in children with demyelinating syndromes. JAMA Neurol. 2020;77(1):82-93. doi:10.1001/jamaneurol.2019.2940 31545352 PMC6763982

[3] Armangue T, Olivé-Cirera G, Martínez-Hernandez E, ; Spanish Pediatric Anti-MOG Study Group. Associations of paediatric demyelinating and encephalitic syndromes with myelin oligodendrocyte glycoprotein antibodies: a multicentre observational study. Lancet Neurol. 2020;19(3):234-246. doi:10.1016/S1474-4422(19)30488-0 32057303

[4] Ramanathan S, Mohammad S, Tantsis E, ; Australasian and New Zealand MOG Study Group. Clinical course, therapeutic responses and outcomes in relapsing MOG antibody-associated demyelination. J Neurol Neurosurg Psychiatry. 2018;89(2):127-137. doi:10.1136/jnnp-2017-316880 29142145 PMC5800335

[5] Baumann M, Grams A, Djurdjevic T, . MRI of the first event in pediatric acquired demyelinating syndromes with antibodies to myelin oligodendrocyte glycoprotein. J Neurol. 2018;265(4):845-855. doi:10.1007/s00415-018-8781-3 29423614

[6] Thomas T, Branson HM, Verhey LH, . The demographic, clinical, and magnetic resonance imaging (MRI) features of transverse myelitis in children. J Child Neurol. 2012;27(1):11-21. doi:10.1177/0883073811420495 21968984

[7] Banwell B, Tenembaum S, Lennon VA, . Neuromyelitis optica-IgG in childhood inflammatory demyelinating CNS disorders. Neurology. 2008;70(5):344-352. doi:10.1212/01.wnl.0000284600.80782.d5 18094334

[8] Elrick MJ, Gordon-Lipkin E, Crawford TO, . Clinical subpopulations in a sample of North American children diagnosed with acute flaccid myelitis, 2012-2016. JAMA Pediatr. 2019;173(2):134-139. doi:10.1001/jamapediatrics.2018.4890 30500056 PMC6439600

[9] Tantsis EM, Prelog K, Alper G, ; Paediatric Myelitis MRI Study Group. Magnetic resonance imaging in enterovirus-71, myelin oligodendrocyte glycoprotein antibody, aquaporin-4 antibody, and multiple sclerosis-associated myelitis in children. Dev Med Child Neurol. 2019;61(9):1108-1116. doi:10.1111/dmcn.14114 30537075

[10] Thompson AJ, Banwell BL, Barkhof F, . Diagnosis of multiple sclerosis: 2017 revisions of the McDonald criteria. Lancet Neurol. 2018;17(2):162-173. doi:10.1016/S1474-4422(17)30470-2 29275977

[11] Mariano R, Flanagan EP, Weinshenker BG, Palace J. A practical approach to the diagnosis of spinal cord lesions. Pract Neurol. 2018;18(3):187-200. doi:10.1136/practneurol-2017-001845 29500319

[12] Fadda G, Brown RA, Longoni G, ; Canadian Pediatric Demyelinating Disease Network. MRI and laboratory features and the performance of international criteria in the diagnosis of multiple sclerosis in children and adolescents: a prospective cohort study. Lancet Child Adolesc Health. 2018;2(3):191-204. doi:10.1016/S2352-4642(18)30026-9 30169254

[13] Krupp LB, Banwell B, Tenembaum S; International Pediatric MS Study Group. Consensus definitions proposed for pediatric multiple sclerosis and related disorders. Neurology. 2007;68(16)(suppl 2):S7-S12. doi:10.1212/01.wnl.0000259422.44235.a8 17438241

[14] Kim SH, Mealy MA, Levy M, . Racial differences ineuromyelitis optica spectrum disorder. Neurology. 2018;91(22):e2089-e2099. doi:10.1212/WNL.0000000000006574 30366977 PMC6282238

[15] Kister I, Bacon T, Cutter GR. How multiple sclerosis symptoms vary by age, sex, and race/ethnicity. Neurol Clin Pract. 2021;11(4):335-347. doi:10.1212/CPJ.0000000000001105 34476125 PMC8382423

[16] O’Mahony J, Marrie RA, Laporte A, . Recovery from central nervous system acute demyelination in children. Pediatrics. 2015;136(1):e115-e123. doi:10.1542/peds.2015-0028 26034241

[17] Waters P, Woodhall M, O’Connor KC, . MOG cell-based assay detects non-MS patients with inflammatory neurologic disease. Neurol Neuroimmunol Neuroinflamm. 2015;2(3):e89. doi:10.1212/NXI.0000000000000089 25821844 PMC4370386

[18] Dubey D, Pittock SJ, Krecke KN, . Clinical, radiologic, and prognostic features of myelitis associated with myelin oligodendrocyte glycoprotein autoantibody. JAMA Neurol. 2019;76(3):301-309. doi:10.1001/jamaneurol.2018.4053 30575890 PMC6440233

[19] Lechner C, Baumann M, Hennes EM, . Antibodies to MOG and AQP4 in children with neuromyelitis optica and limited forms of the disease. J Neurol Neurosurg Psychiatry. 2016;87(8):897-905. doi:10.1136/jnnp-2015-311743 26645082

[20] Mariotto S, Ferrari S, Monaco S, . Clinical spectrum and IgG subclass analysis of anti-myelin oligodendrocyte glycoprotein antibody-associated syndromes: a multicenter study. J Neurol. 2017;264(12):2420-2430. doi:10.1007/s00415-017-8635-4 29063242 PMC5688213

[21] Wang C, Narayan R, Greenberg B. Anti-myelin oligodendrocyte glycoprotein antibody associated with gray matter predominant transverse myelitis mimicking acute flaccid myelitis: a presentation of two cases. Pediatr Neurol. 2018;86:42-45. doi:10.1016/j.pediatrneurol.2018.06.003 30077551

[22] Rabasté S, Cobo-Calvo A, Nistiriuc-Muntean V, . Diagnostic value of bright spotty lesions on MRI after a first episode of acute myelopathy. J Neuroradiol. 2021;48(1):28-36. doi:10.1016/j.neurad.2020.04.006 32407908

[23] Yonezu T, Ito S, Mori M, . “Bright spotty lesions” on spinal magnetic resonance imaging differentiate neuromyelitis optica from multiple sclerosis. Mult Scler. 2014;20(3):331-337. doi:10.1177/1352458513495581 23828869

[24] Hyun JW, Kim SH, Jeong IH, Lee SH, Kim HJ. Bright spotty lesions on the spinal cord: an additional MRI indicator of neuromyelitis optica spectrum disorder? J Neurol Neurosurg Psychiatry. 2015;86(11):1280-1282. doi:10.1136/jnnp-2014-309761 25575845

[25] Mohseni SH, Skejoe HPB, Wuerfel J, . Leptomeningeal and intraparenchymal blood barrier disruption in a MOG-IgG-positive patient. Case Rep Neurol Med. 2018;2018:1365175. doi:10.1155/2018/1365175 30834146 PMC6374805

[26] Rinaldi S, Davies A, Fehmi J, ; Australian and New Zealand MOG Study Group. Overlapping central and peripheral nervous system syndromes in MOG antibody-associated disorders. Neurol Neuroimmunol Neuroinflamm. 2020;8(1):e924. doi:10.1212/NXI.0000000000000924 33272955 PMC7803332

[27] Netravathi M, Holla VV, Nalini A, . Myelin oligodendrocyte glycoprotein-antibody-associated disorder: a new inflammatory CNS demyelinating disorder. J Neurol. 2021;268(4):1419-1433. doi:10.1007/s00415-020-10300-z 33188477

[28] Budhram A, Kunchok AC, Flanagan EP. Unilateral leptomeningeal enhancement in myelin oligodendrocyte glycoprotein immunoglobulin G-associated disease. JAMA Neurol. 2020;77(5):648-649. doi:10.1001/jamaneurol.2020.0001 32119057

[29] Budhram A, Mirian A, Le C, Hosseini-Moghaddam SM, Sharma M, Nicolle MW. Unilateral cortical FLAIR-hyperintense lesions in anti-MOG-associated encephalitis with seizures (FLAMES): characterization of a distinct clinico-radiographic syndrome. J Neurol. 2019;266(10):2481-2487. doi:10.1007/s00415-019-09440-8 31243540

[30] Cobo-Calvo A, Ruiz A, Maillart E, ; OFSEP and NOMADMUS Study Group. Clinical spectrum and prognostic value of CNS MOG autoimmunity in adults: the MOGADOR study. Neurology. 2018;90(21):e1858-e1869. doi:10.1212/WNL.0000000000005560 29695592

[31] Banks SA, Morris PP, Chen JJ, . Brainstem and cerebellar involvement in MOG-IgG-associated disorder versus aquaporin-4-IgG and MS. J Neurol Neurosurg Psychiatry. 2021;92(4):384-390. doi:10.1136/jnnp-2020-325121 33372052 PMC8592388

[32] Okumura A, Mori H, Fee Chong P, ; Acute Flaccid Myelitis Collaborative Study Investigators. Serial MRI findings of acute flaccid myelitis during an outbreak of enterovirus D68 infection in Japan. Brain Dev. 2019;41(5):443-451. doi:10.1016/j.braindev.2018.12.001 30594353

[33] Nakamura T, Kaneko K, Watanabe G, . Myelin oligodendrocyte glycoprotein-IgG-positive, steroid-responsive combined central and peripheral demyelination with recurrent peripheral neuropathy. Neurol Sci. 2021;42:1135-1138. doi:10.1007/s10072-020-04822-7 33078247

[34] Sundaram S, Nair SS, Jaganmohan D, Unnikrishnan G, Nair M. Relapsing lumbosacral myeloradiculitis: an unusual presentation of MOG antibody disease. Mult Scler. 2020;26(4):509-511. doi:10.1177/1352458519840747 30931808

[35] Vazquez Do Campo R, Stephens A, Marin Collazo IV, Rubin DI. MOG antibodies in combined central and peripheral demyelination syndromes. Neurol Neuroimmunol Neuroinflamm. 2018;5(6):e503. doi:10.1212/NXI.0000000000000503 30246057 PMC6147156

[36] Asnafi S, Morris PP, Sechi E, . The frequency of longitudinally extensive transverse myelitis in MS: a population-based study. Mult Scler Relat Disord. 2020;37:101487. doi:10.1016/j.msard.2019.101487 31707235

[37] López-Chiriboga AS, Majed M, Fryer J, . Association of MOG-IgG serostatus with relapse after acute disseminated encephalomyelitis and proposed diagnostic criteria for MOG-IgG-associated disorders. JAMA Neurol. 2018;75(11):1355-1363. doi:10.1001/jamaneurol.2018.1814 30014148 PMC6248120

[38] Sechi E, Buciuc M, Pittock SJ, . Positive predictive value of myelin oligodendrocyte glycoprotein autoantibody testing. JAMA Neurol. 2021;78(6):741-746. doi:10.1001/jamaneurol.2021.0912 33900394 PMC8077043

[39] Mariano R, Messina S, Kumar K, Kuker W, Leite MI, Palace J. Comparison of clinical outcomes of transverse myelitis among adults with myelin oligodendrocyte glycoprotein antibody vs aquaporin-4 antibody disease. JAMA Netw Open. 2019;2(10):e1912732. doi:10.1001/jamanetworkopen.2019.12732 31596489 PMC6802235

[40] Mariano R, Messina S, Roca-Fernandez A, Leite MI, Kong Y, Palace JA. Quantitative spinal cord MRI in MOG-antibody disease, neuromyelitis optica and multiple sclerosis. Brain. 2021;144(1):198-212. doi:10.1093/brain/awaa347 33206944

[41] Cobo-Calvo Á, Sepúlveda M, Bernard-Valnet R, . Antibodies to myelin oligodendrocyte glycoprotein in aquaporin 4 antibody seronegative longitudinally extensive transverse myelitis: clinical and prognostic implications. Mult Scler. 2016;22(3):312-319. doi:10.1177/1352458515591071 26209592

[42] Jurynczyk M, Geraldes R, Probert F, . Distinct brain imaging characteristics of autoantibody-mediated CNS conditions and multiple sclerosis. Brain. 2017;140(3):617-627. doi:10.1093/brain/aww350 28364548

