# Supplementary material for: Comparison of Spinal Cord Magnetic Resonance Imaging Features Among Children With Acquired Demyelinating Syndromes
Source: JAMA Netw Open. 2021 Oct 13;4(10):e2128871. doi: 10.1001/jamanetworkopen.2021.28871 (PMC8515204; doi:10.1001/jamanetworkopen.2021.28871)
Supplement: Supplement 1. — eAppendix. Spine MRI Scoring Tool eTable 1. Features Associated With LETM eTable 2. Spine Imaging Features of ADEM vs Non-ADEM MOGAD eFigure 1. MOGAD With Clinical Presentation of Myelitis and Initial Negative Spine MRI Findings eFigure 2. Sagittal and Axial Postgadolinium T1 MR Imaging of the Spine of 5 Different Children With MOGAD Demonstrating Leptomeningeal and Root Enhancement eFigure 3. LETM in a 14-Year-Old Girl With MS [file jamanetwopen-e2128871-s001.pdf]

Supplemental Online Content

Fadda G, Alves CA, O’Mahony J, et al; Canadian Pediatric Demyelinating Disease Study Group. Comparison of spinal cord magnetic resonance imaging features among children with acquired demyelinating syndromes. *JAMA Netw Open*. 2021;4(10):e2128871. doi:10.1001/jamanetworkopen.2021.28871

eAppendix. Spine MRI Scoring Tool
eTable 1. Features Associated With LETM
eTable 2. Spine Imaging Features of ADEM vs Non-ADEM MOGAD
eFigure 1. MOGAD With Clinical Presentation of Myelitis and Initial Negative Spine MRI Findings
eFigure 2. Sagittal and Axial Postgadolinium T1 MR Imaging of the Spine of 5 Different Children With MOGAD Demonstrating Leptomeningeal and Root Enhancement
eFigure 3. LETM in a 14-Year-Old Girl With MS

This supplemental material has been provided by the authors to give readers additional information about their work.

© 2021 Fadda G et al. *JAMA Network Open.*

eAppendix: Spine MRI Scoring Tool

General considerations:
All spine MRI scans are initially evaluated for study suitability based on image quality. If the quality of a scan is considered too poor for reliable scoring, this is explicitly reported and the MRI scan is excluded from the analysis.

Each MRI feature is scored as "present" only if the scorer has good degree of certainty. The degree of certainty increased if a particular lesion/feature is clearly seen in two different views.
Any lesion deemed "uncertain" by the initial scorer is flagged for independent adjudication by the most senior neuroradiologist MRI reviewer (AV).

Lesions are always counted in a cranio-caudal order.

Multiple features and signs can be assigned to the same lesion, acknowledging that they can occur simultaneously at the same level but in different views (e.g. snake eyes and pencil signs) or at different levels of the same lesion.

The adjudication of some features (e.g. "tumefactive lesion") is recognized as being highly subjective in its nature.

Operational definitions:
All parameters are binary (ie, scored as present or absent)
- Lesion present: area of clear focal hyperintensity on a T2-weighted image.
- Cervical lesion: lesions involving the spinal cord between the occipital foramen to the level of C7 (included).
- Thoracic lesion: lesions involving the spinal cord between the level of T1 to the lumbar enlargement.
- Lumbar lesion: lesion involving the lumbar enlargement or caudal to it. Conal and lumbar locations are equivalent.
- Cervicomedullary junction lesion: Lesion extended cranially to involve the medulla (to the level of the olive and obex)
- Longitudinally extensive lesion: a single spinal cord lesion extending over 3 vertebral segments or more. For any suspected longitudinally extensive lesion detected on the sagittal plane, the presence of contiguous short lesions should be ruled out by analysis of axial plane images.
- Tumefactive lesion: Increase of the diameter of the spine in correspondence to a lesion compared to the above and below segments. Caution should be applied in correspondence of the thoracic and lumbar physiological enlargement.

Features to be assessed on axial views:

- **Anterior lesion:** Lesion involving the ventral columns and/or ventral horns.
- **Lateral lesion:** Lesion involving the lateral columns
- **Posterior lesion:** lesion involving the dorsal columns and/or dorsal horns.
- **Involvement of central gray:** lesion involving the central grey matter.
- **Involvement of gray and white matter:** lesion simultaneously involving portion of both spinal cord white and grey matter.
- **Involvement of only white matter:** lesion involving exclusively white matter tracts.
- **Involvement of only gray matter:** lesion involving exclusively the spinal cord grey matter.
- **Involvement of complete cross-section:** involvement of all gray matter and most of white matter in at least one slice. Presence of small areas of normal appearing tissue is accepted.
- **H sign**: hyperintensity on T2-weighted images restricted to spinal cord gray matter resulting in a T2-hyperintense H shaped area. Cases in which the involvement of the gray matter is clearly predominant are

© 2021 Fadda G et al. *JAMA Network Open.*

scored as positive for H sign, allowing for additional involvement of the surrounding WM. Determination of increased intensity of the central gray matter on gradient echo sequences (rather than standard axial T2 sequences) requires careful adjudication owing to the fact that gray can naturally appear hyperintense/bright on these sequences.

- **Snake-eyes sign**: bilaterally symmetric circular to ovoid foci of high T2-weighted signals in the anterior horns of the spinal cord gray matter.
- **Bright spot**: T2 hyperintensity greater than surrounding CSF without flow void effects

Features to be assessed in post-contrast images:

- **Nodular enhancement:** nodular hyperintense signal on T1-weighted contrast-enhanced imaging corresponding to an area of hyperintensity in T2 -weighted images
- **Ring enhancement:** ring-like hyperintense signal on T1-weighted contrast-enhanced imaging (not hyperintense on T1-weighted pre-contrast imaging) corresponding to an area of hyperintensity in T2 -weighted images
- **Leptomeningeal enhancement:** linear or nodular hyperintensity on T1-weighted contrast-enhanced imaging corresponding anatomically to arachnoid and pia mater.
- **Root enhancement:** hyperintensity of spinal roots on T1-weighted contrast-enhanced imaging compared to pre-contrast T1-weighted images.

Non-binary parameters:
Lesion length: number of spinal vertebral body segments spanned by the lesion.
Number of lesions: number of individual lesions detected in a given scan

© 2021 Fadda G et al. *JAMA Network Open.*

eTable 1: Features Associated With LETM

	MOGAD	MS	Seronegative myelitis	p value (MOG vs MS)	p value (MOG vs Seroneg. myelitis)
**Participants with LETM**	30/40 (75%)	1/21 (5%)	20/46 (43%)	<0.0001	0.00024
**Phenotype at scan TM**	8 (27%)	1 (100%)	14 (70%)	0.29	0.74
**Phenotype at scan ADEM**	5 (17%)	0 (0%)	4 (20%)	1	1
**Phenotype at scan ADEM + TM**	10 (33%)	0 (0%)	1 (5%)	1	0.033
**Phenotype at scan ON+TM**	3 (10%)	0 (0%)	0 (0%)	1	0.27
**Other phenotype at scan**	4 (13%)	0	1 (5%)	1	0.64
**Involvement of C spine**	27 (90%)	1 (100%)	16 (80%)	1	0.42
**Involvement of T spine**	24 (80%)	0 (0%)	15 (75%)	0.23	0.94
**Involvement of L spine**	22 (73%)	0 (0%)	13 (65%)	0.29	0.75
**Maximum length LETM (median,IQR,range)**	>10 (6.5->10),(3.50->10)	3.00	>10 (5.5->10),(4.00->10)	0.038	0.36
**Nodular enhancement**	1/24 (4%)	1/1 (100%)	0/17 (0%)	0.08	1
**Leptomeningeal enhancement**	19/24 (79%)	0/1 (0%)	6/17 (35%)	0.24	0.012
**Root enhancement**	9/24 (38%)	0/1 (0%)	4/17 (24%)	1	0.5

Data are reported as n (%) or as a median (IQR),(range).

© 2021 Fadda G et al. *JAMA Network Open.*

eTable 2: Spine Imaging Features of ADEM vs Non-ADEM MOGAD

	ADEM	Non-ADEM	p value
**Participants**	17	23	1
**Sex (Female)**	11 (65%)	12 (52%)	0.64
**Age at disease onset**	4.91 (3.15-6.90)	8.69 (5.10-12.41)	0.028
**Symptoms at scan**	17 (100%)	18 (78%)	0.061
**Brain Lesions**	17/17 (100%)	11/23 (48%)	0.00029
**Total spine lesion count**	2.00 (1.00-2.00)	1.00 (1.00-2.00)	0.24
**Cervical lesion**	11/17 (65%)	19/23 (83%)	0.27
**Thoracic lesion**	13/16 (81%)	18/23 (78%)	1
**Lumbar lesion**	11/16 (69%)	14/23 (61%)	0.87
**Any LETM**	13/17 (76%)	17/23 (74%)	0.99
**Length LETM**	11 (7-11)	11 (6-11)	0.44
**Any gray and white involv.**	14/15 (93%)	16/20 (80%)	0.36
**Any only white matter involv.**	0/15 (0%)	2/20 (10%)	0.5
**Any only gray matter involv.**	3/15 (20%)	4/20 (20%)	1
**Any Complete Cross-section**	11/15 (73%)	14/20 (70%)	1
**Any H sign**	10/15 (67%)	12/20 (60%)	0.96
**Any snake eyes**	4/15 (27%)	2/20 (10%)	0.37
**Any tumefactive lesion**	5/17 (29%)	9/23 (39%)	0.76
**Any Nodular Enhancement**	3/13 (23%)	5/19 (26%)	1
**Any Complete Ring Enhancement**	0/13 (0%)	0/19 (0%)	1
**Any Leptomeningeal Enhancement**	8/13 (62%)	17/19 (89%)	0.091
**Any Root Enhancement**	5/13 (38%)	6/19 (32%)	0.98
**Lesion Resolution**	7/9 (77%)	6/11 (55%)	0.37
**EDSS at 5 years from presentation**	0.00 (0.00-0.25)	1.00 (0.00-1.50)	0.08

Data are reported as n (%) or as a median (IQR).

© 2021 Fadda G et al. *JAMA Network Open.*

eFigure 1: MOGAD With Clinical Presentation of Myelitis and Initial Negative Spine MRI Findings.

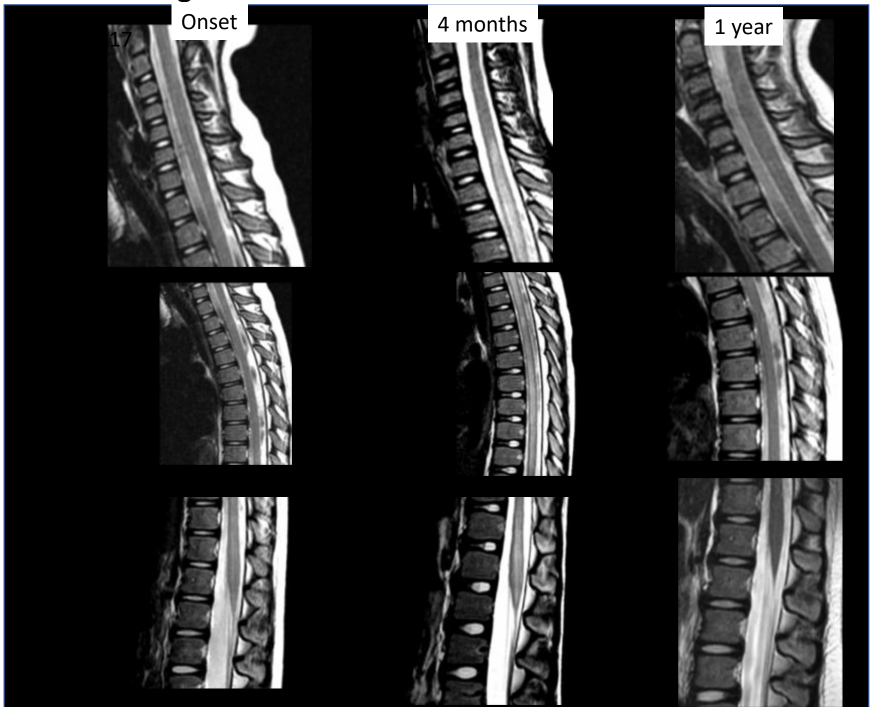

Normal sagittal T2 spine MRI 15 days from clinical presentation with myelitis (onset). Detection of LETM with more than 10 vertebral bodies of extension at 4 months in the absence of new symptoms, and complete lesion resolution at the subsequent follow-up (1 year from onset)

eFigure 2: Sagittal and Axial Postgadolinium T1 MR Imaging of the Spine of 5 Different Children With MOGAD Demonstrating Leptomeningeal and Root Enhancement.

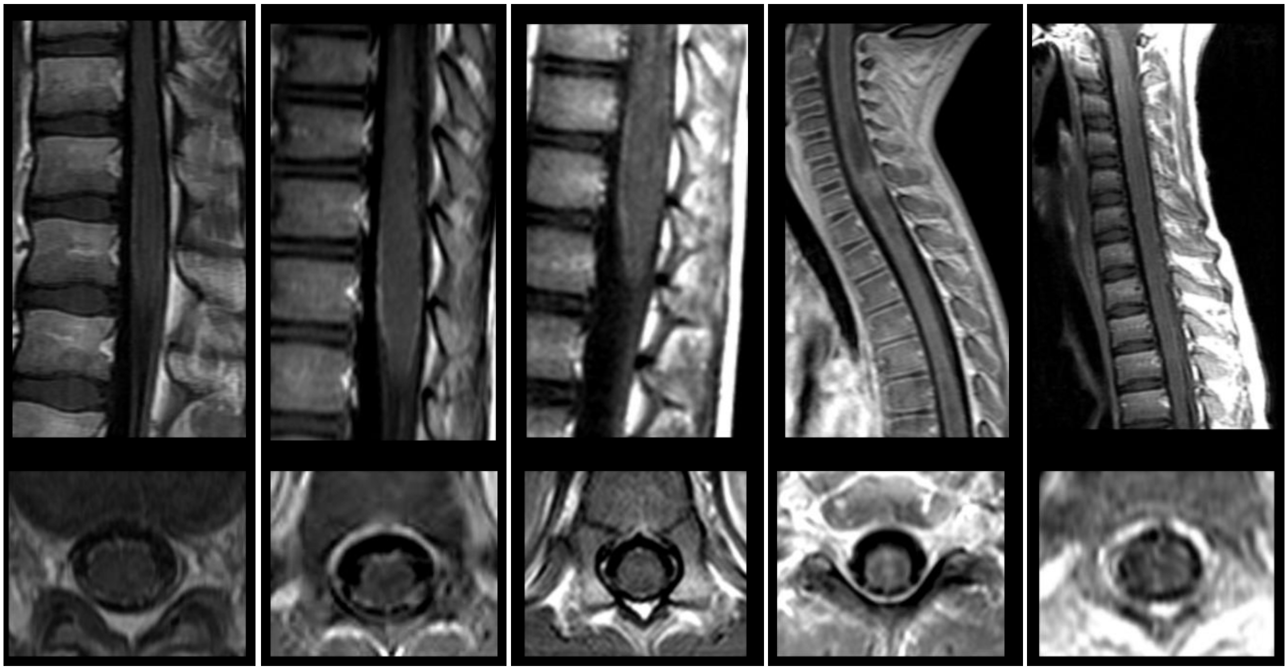

© 2021 Fadda G et al. *JAMA Network Open.*

eFigure 3: LETM in a 14-Year-Old Girl With MS.

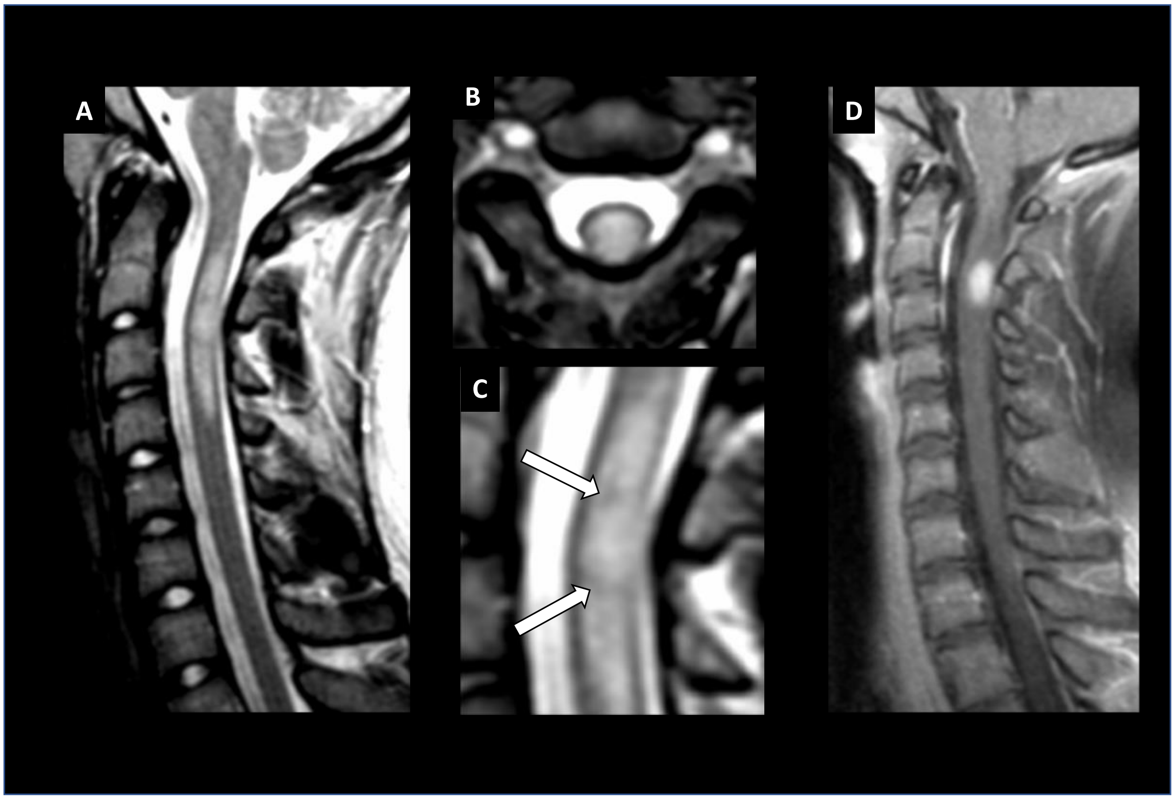

Sagittal and axial T2 MR imaging of the cervical spine (A-C) and sagittal post-gadolinium T1 (D) demonstrating a lesion extending over 3 vertebral segments (A). The lesion presents a central short core of T2 hyperintensity (arrows, C), associated with intense contrast enhancement (D).

© 2021 Fadda G et al. *JAMA Network Open.*
