# Supplementary material for: Comparison of Spinal Cord Magnetic Resonance Imaging Features Among Children With Acquired Demyelinating Syndromes
Source: JAMA Netw Open. 2021 Oct 13;4(10):e2128871. doi: 10.1001/jamanetworkopen.2021.28871 (PMC8515204; doi:10.1001/jamanetworkopen.2021.28871)
Supplement: Supplement 2. — Canadian Pediatric Demyelinating Disease Study Group [file jamanetwopen-e2128871-s002.pdf]

Supplemental Online Content: Nonauthor Collaborators

*Indicates required information. Only first name, last name, and suffix will appear in PubMed.

*Group Name(s): Canadian Pediatric Demyelinating Disease Study Group							
***First Name and Middle Initial(s)**	***Last Name**	***Suffix (eg, Jr, III)**	Academic Degrees	Institution	Location (city, state/province, country)	Role or Contribution, eg, chair, principal investigator	Group (if more than 1 Group listed in the byline) and/or Subgroup (eg, Steering Committee)
Brenda	Banwell		MD	Children Hospital of Philadelphia	Philadelphia, PA, USA	principal investigator	
Amit	Bar-Or		MD	University of Pennsylvania	Philadelphia, PA, USA	principal investigator	
Douglas L.	Arnold		MD	McGill Unversity	Montreal, QC, Canada	principal investigator	
Ruth Ann	Marrie		MD, PhD	University of Mannitoba	Winnipeg, MB, Canada	principal investigator	
E. Ann	Yeh		MD	University of Toronto	Toronto, ON, Canada	principal investigator	
Mark	Awuku		MD	University of Windsor	Windsor, ON, Canada	sub-site investigator	
J. Burke	Baird		MD	McMaster University	Hamilton, ON, Canada	sub-site investigator	
Virender	Bhan		MD	Dalhousie University	Halifax, NS, Canada	sub-site investigator	
David	Buckley		MD	Janeway Children’s Health and Rehabilitation Centre	St John, NB, Canada	sub-site investigator	
David	Callen		MD	Hamilton Health Sciences Center	Hamilton, ON, Canada	sub-site investigator	
Mary B.	Connolly		MBBCh	Children’s Hospital of British Columbia	Vancouver, BC, Canada	sub-site investigator	
Marie-Emmanuelle	Dilenge		MD	Montreal Children's Hospital	Montreal, QC, Canada	sub-site investigator	
Asif	Doja		MD	Children’s Hospital of Eastern Ontario	Ottawa, ON, Canada	sub-site investigator	
Simon	Levin		MD	University Hospital London	London, ON, Canada	sub-site investigator	
Anne	Lortie		MD	CHU Sainte-Justine	Montreal, QC, Canada	sub-site investigator	
E. Athen	MacDonald		MD	Hôtel-Dieu de Paris	Kingston, ON, Canada	sub-site investigator	
Jean K.	Mah		MD	Alberta Children’s Hospital	Calgary, AL, Canada	sub-site investigator	
Brandon	Meaney		MD	Hamilton Health Sciences Center	Hamilton, ON, Canada	sub-site investigator	
David	Meek		MD	St John Regional Hospital Facility	St John, NB, Canada	sub-site investigator	
Daniela	Pohl		MD	Children’s Hospital of Eastern Ontario	Ottawa, ON, Canada	sub-site investigator	
Guillaume	Sebire		MD	Montreal Children's Hospital	Montreal, QC, Canada	sub-site investigator	
Sunita	Venkateswaran		MD	Children’s Hospital of Eastern Ontario	Ottawa, ON, Canada	sub-site investigator	

Supplemental Online Content: Nonauthor Collaborators

*Indicates required information. Only first name, last name, and suffix will appear in PubMed.

***First Name and Middle Initial(s)**	***Last Name**	***Suffix (eg, Jr, III)**	Academic Degrees	Institution	Location (city, state/province, country)	Role or Contribution, eg, chair, principal investigator	Group (if more than 1 Group listed in the byline) and/or Subgroup (eg, Steering Committee)
Amy	Waldman		MD	Children Hospital of Philadelphia	Philadelphia, PA, USA	sub-site investigator	
Katherine	Wambera		MD	Victoria General Hospital	Victoria, BC, Canada	sub-site investigator	
Ellen	Wood		MD	Dalhousie University	Halifax, NS, Canada	sub-site investigator	
Jerome	Yager		MD	Children's Stollery Hospital	Edmonton, AL, Canada	sub-site investigator	
